# Acephate Exposure Induces Transgenerational Ovarian Developmental Toxicity by Altering the Expression of Follicular Growth Markers in Female Rats

**DOI:** 10.3390/biology13121075

**Published:** 2024-12-20

**Authors:** Abeer Alhazmi, Saber Nahdi, Saleh Alwasel, Abdel Halim Harrath

**Affiliations:** Department of Zoology, College of Science, King Saud University, Riyadh P.O. Box 145111, Saudi Arabia

**Keywords:** organophosphate, acephate, ovarian toxicity, transgenerational inheritance, female infertility

## Abstract

Acephate is an organophosphate foliar and soil insecticide that is used worldwide. In this study, the transgenerational ovarian developmental toxicity caused by acephate, along with its in utero reprogramming mechanisms, were explored. Thirty female virgin Wistar albino rats were randomly assigned to three groups: one control group and two acephate treatment groups. The treatment groups received daily low or high doses of acephate (34.2 mg/kg or 68.5 mg/kg body weight, respectively) from gestational day 6 until spontaneous labor, resulting in F1 offspring. At 28 days, a subgroup of F1 females were euthanized. The ovaries were extracted, thoroughly cleaned, and weighed before being fixed for further analysis. The remaining F1 females were mated with normal males to produce the F2 generation. The F1 female offspring presented reduced fertility and body weight, whereas the ovarian weight index and sex ratio increased in a dose-dependent manner. Structural analysis revealed altered follicular abnormalities with ovarian cells displaying pyknotic nuclei. Additionally, the gene and protein expression of *Cyp19* decreased, whereas that of *Gdf-9* increased in the high-dose treatment group (68.5 mg/kg). We also observed significantly increased expression levels of ovarian estrogen receptor 1 (*Esr1*) and insulin-like growth factor 1 (*Igf1*), whereas *Insl3* expression was significantly decreased. The F2 female offspring presented reproductive phenotype alterations similar to those of F1 females including decreased fertility, reduced *Cyp19* gene and protein expression, and structural ovarian abnormalities similar to those of polycystic ovary syndrome (PCOS). In conclusion, acephate induced ovarian developmental toxicity across two generations of rats, which may be linked to changes in the ovarian *Cyp19*, *Gdf9*, *Insl3*, and *Igf1* levels.

## Simple Summary

This study investigated the harmful effects of acephate, a commonly used pesticide, on the reproductive well-being of female rats. We found that when pregnant rats were exposed to acephate, their offspring experienced significant reproductive health issues over two generations. These issues are caused by irregularities in ovarian structure, reduced fertility, and changes in the expression of key genes and proteins involved in ovarian cell growth and differentiation. These effects were also observed in the second generation, even though they were not directly exposed to the pesticide, suggesting that the damage can be inherited from one generation to another. Notably, high doses of acephate caused more severe effects including ovarian cysts and disrupted aromatase enzyme regulation, responsible for estrogen production. The findings of this research highlight the potential long-term risks of pesticide exposure not only for individuals directly exposed, but also for future generations. They emphasize the need for stricter regulations on pesticide use and raised awareness about the impact of environmental pollutants on female reproductive health. Such knowledge is valuable for informing public health policies and encouraging safer agricultural practices to reduce harm to human and animal reproductive systems.

## 1. Introduction

According to recent epidemiological research, exposure to environmental pollutants throughout one’s lifetime increases the chance of contracting chronic diseases including cancer [1,2], diabetes [3], heart disease [3,4], obesity [5], neurological disorders [5], and reproductive toxicity [6,7,8,9,10]. Indeed, worldwide, exposure to chemical pollutants has increased by approximately 35% in the previous two decades [11], and unhealthy environmental conditions have been reported to be the cause of 12.6 million fatalities annually [12].

Among these pollutants, pesticides are either natural or synthetic substances utilized to manage plant diseases and pests. They can be categorized into three main types on the basis of their application: herbicides, insecticides, and fungicides [13,14]. These toxins are now causing many concerns since they have substantial and enduring impacts on organisms and are linked to several diseases including cancer [15,16,17]. Indeed, pesticide residues remain in the soil after application and can be transferred to and accumulate in the air, crops, and animals [18,19]. Prolonged exposure to pesticides has been linked to several malignancies including lung, breast, lymphoma, leukemia, prostate, brain, colon, stomach, and liver malignancies [20,21]. One of the most popular pesticides is the organophosphate (OP) compound, which has a wide range of effectiveness against insects and other pests [22]. Two of the most popular and effective OPs used for pest management in agriculture are acephate and methamidophos [23,24,25]. Methamidophos is an acetylcholinesterase (AChE) inhibitor, and acephate metabolizes into its intermediate methamidophos [26,27]. Acephate is used in horticulture and fruit, vegetable, vine, and hop production to manage a variety of sucking and chewing insect species, particularly aphids including resistant species. In addition, it manages grass, mint, trees used in forestry, lepidopteran larvae, sawflies, and thrips in the aforementioned crops [28]. This natural pollutant triggers an overproduction of reactive oxygen species (ROS), which can lead to various adverse health issues such as diabetes and reproductive toxicity [3,6].

Interestingly, in utero exposure to environmental contaminants can harm the developing fetus, potentially leading to lifelong disorders in adulthood, according to recent experimental and epidemiological research [7,9,29,30]. In particular, chronic metabolic dysfunction caused by the ingestion of pollutants such as pesticides has been linked to metabolic reprogramming [1]. Prenatal pesticide exposure has been linked to kidney diseases, obesity, and neurobehavioral disorders in rats and humans [2,3,15,17,31]. Similar studies have also shown that exposure to organophosphate pesticides may harm the gonadal and endocrine systems of offspring [21,32,33]. Thus, the use of emerging environmental pollutants as risk factors for female infertility is increasing daily [34]. Indeed, because environmental pollutants are now linked to the development of illnesses in both wildlife and humans [35,36], the reproductive health of both men and women may be negatively impacted by exposure to pollutants, particularly endocrine-disrupting chemicals [37]. Consequently, the principal goal of our study was to examine the transgenerational inheritance impacts of acephate exposure on ovarian function during prenatal development in female offspring of two generations of rats, F1 and F2. This is the first study to demonstrate how in utero acephate exposure impacts ovarian function in adult rat offspring by exploring the histopathology of ovarian tissue and the expression of folliculogenesis- and steroidogenesis-related markers.

## 2. Materials and Methods

### 2.1. Design of Study and Sampling

This research received approval from the Scientific Research Ethics Committee (Reference No: KSU-SE-24-13) at King Saud University in Riyadh, Saudi Arabia. It was conducted following the established guidelines. All the experimental protocols adhered to and complied with the Animal Research: Reporting of In Vivo Experiments (ARRIVE) guidelines. Thirty 60-day-old virgin female Wistar albino rats, each weighing between 210 and 240 g, were housed individually in a controlled environment. The room was maintained at 21 °C with a reversed light–dark cycle, and the rats were given unrestricted access to food and water. After being weighed, the rats were randomly divided into three treatment groups. First, the control group (n = 10) received only distilled water, while the second and third groups (n = 10 rats each) were administered acephate dissolved in distilled water (98% purity; Sigma–Aldrich, St. Louis, MO, USA) via gavage starting on day 6 of gestation, which corresponds to the time of fetal implantation, and continued until birth. Groups 2 and 3 were given acephate doses of 34.4 mg/kg and 68.5 mg/kg body weight, respectively, on the basis of oral LD50 values of 1/60 and 1/30, according to the reported LD50 of acephate (866 mg/kg) and its known harmful impacts on biological systems [17]. The first-generation (F1) animals wee the offspring we acquired from mothers who received acephate treatment after parturition, whereas the control group was known as the first-generation control group (CF1). Body weight measurements of the newborns across the different groups and generations were obtained by calculating the average weight of the siblings for each female, resulting in 10 values per group. The sex ratio was determined by calculating the ratio of the number of males to the number of females.

Before puberty, when they reached 4 weeks of age, a portion of the female F1 and CF1 offspring (10 females from each group) were euthanized using a transparent plastic chamber connected to a carbon dioxide tube, delivering a flow rate of 10 L/h for 10 min. The ovaries were quickly removed, cleaned, weighted, and fixed in the corresponding fixative (10% neutral buffer formalin (NBF) or RNAlater; Qiagen, Westburg, The Netherlands). The ovary weight index was calculated as follows: average weight of the two ovaries divided by the weight of the corresponding female.

After being allowed to reach sexual maturity, the surviving F1 and CF1 female rats were mated with healthy male rats. The F1 female rats treated with acephate gave birth to a second generation of offspring (F2), whereas the control group rats produced a separate control subgroup for the second generation (CF2). The ovaries of the F2 and CF2 female offspring were sampled, and the offspring were euthanized when they were 4 weeks old.

The ovary weight index and fertility rate were used to compare the reproductive capacity of the groups among the two generations F1 and F2.

### 2.2. Histopathology

The ovary tissues were fixed in 10% neutral buffered formalin for 2 h, dehydrated and embedded in paraffin blocks. Sections (5–7 μm thick) were placed on glass slides with warm (30 °C) water and albumin-cerol fixative to aid in adherence and dried on a hotplate. After the wrinkles were removed, hematoxylin and eosin (H&E) staining was used to stain the sections [38].

### 2.3. Immunofluorescence Staining and Confocal Microscopy

The tissue slides were placed on a hotplate set at 60 degrees Celsius and cleared twice for ten minutes each time in xylene. Following ethanol rehydration (100% 2 × 7 min, 95% 2 × 7 min, 80% 7 min, 70% 7 min, and 50% 7 min), the samples were rinsed with distilled water and PBS solution. Once the slides were washed, they were dried by placing them diagonally on a table lined with dry paper towels to ensure that all washing fluid was completely removed. To slow the rate at which the solution that would be applied to the tissue sections in the subsequent steps evaporated, the slides were dried and then placed in an appropriate container on the floor, which was covered in layers of paper towels that had been moistened with water. The tissue sections were placed in 0.1% sodium citrate and 0.1% Triton X-100. Nonspecific staining was blocked with a blocking solution for 45 min at room temperature. After the rabbit anti-CYP19 and anti-GDF-9 primary antibodies (1:300 and 1:100, respectively, DGpeptides Co. Ltd., Wuhan, China) were diluted, the slides were incubated in a refrigerator overnight at 4 °C. On the second day, the slides were washed four times with 1× PBS before being exposed to the anti-rabbit secondary antibody (1:500 dilution, Alexa Fluor 488) for 45 min at room temperature in the dark. The slides were first cleaned with TE buffer and PBS, and then Hoechst solution (diluted 0.1:3000) was added. Afterward, the slides were washed with TE buffer, dried, and covered with a coverslip. The edges of the coverslip were sealed with liquid nail polish. We used a Zeiss spinning disk confocal microscope to capture the images [2].

### 2.4. Analysis of Gene Expression

Using a Qiagen RNeasy Mini Kit (Qiagen, Westburg, The Netherlands), the total RNA was extracted from 30 mg of RNA-stabilized tissue, and Qiagen RNase-Free DNase was used for on-column DNase treatment. Using the gene-specific primers provided in Table S1, real-time PCR was carried out via an Applied Biosystems 7500 Fast Real-Time PCR machine (Carlsbad, CA, USA) with SYBR Green. The cDNA of these samples was generated using an iScriptTM cDNA Synthesis Kit (Applied Biosystems, Carlsbad, CA, USA). Several sets of primers along with the cDNA were used to amplify the cDNA for relative quantification via RT–PCR. For every sample, this procedure was carried out in triplicate. Using the 2-DDCt approach, we determined the relative amount of each gene transcript, and normalization was performed using *Gapdh* as a housekeeping gene.

### 2.5. Statistical Analysis

Statistical analysis was conducted via GraphPad Prism version 10. One-way analysis of variance (ANOVA) was conducted, followed by Tukey’s multiple comparison test for statistical analysis. The results were expressed as the mean ± standard deviation (SD). A significance threshold was established at a *p* value of less than 0.05.

## 3. Results

### 3.1. Effect of Acephate on Offspring Body Weight and Sex Ratio

**Figure 1:**
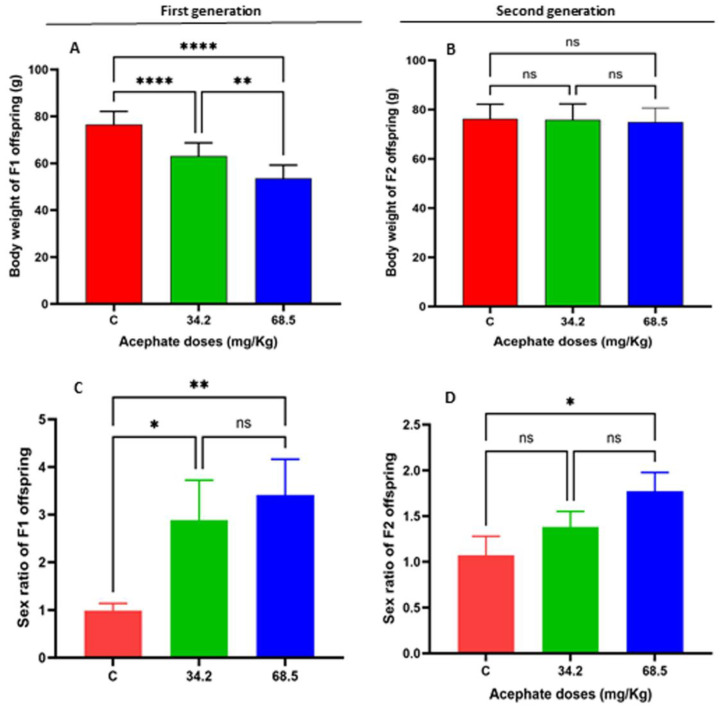
Body weight measurements of both the F1 (**A**) and F2 (**B**) treatment groups were compared with those in the control group. In the first generation, rats treated with either low-dose (34.2 mg/kg) or high-dose (68.5 mg/kg) acephate presented a significant reduction in body weight, whereas the second generation presented no significant changes. Compared with that in the control group, the sex ratio significantly increased in the first generation F1 (**C**). However, in the F2 generation (D), the sex ratio of the low-dose (34.2 mg/kg) treated females did not differ significantly from that of the control group, although the high-dose (68.5 mg/kg) treatment group presented an increased sex ratio compared with the control group. (*) *p* value < 0.05, (**) *p* value < 0.01, (****) *p* value < 0.0001), ns: non signficant.

When the acephate treatment groups were compared with the control groups, the results indicated that acephate caused significant weight loss in the two treatment groups (34.2 and 68.5 mg/kg) of the F1 offspring compared with the CF1 offspring (Figure 1A). However, there was no significant difference in the body weights of the F2 offspring compared with the CF2 offspring (Figure 1B).

The sex ratio of the FI offspring was significantly greater in the two treatment groups (34.2 and 68.5 mg/kg) compared to the CF1 group (Figure 1C), whereas the sex ratio of the F2 offspring was significantly greater only in the high-dose acephate-treated group (68.5 mg/kg) (Figure 1D).

### 3.2. Effects of Acephate on the Ovarian Index and Fertility Rate

**Figure 2:**
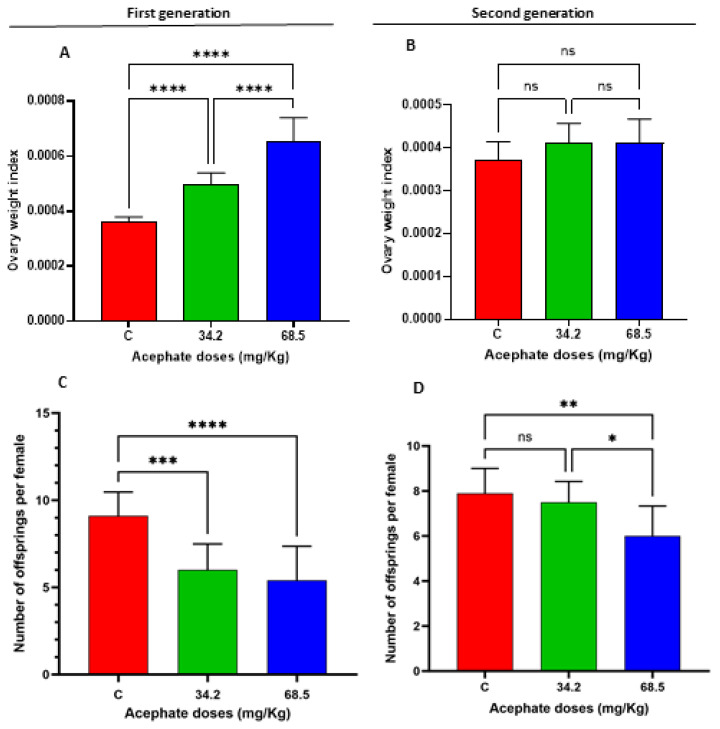
Ovarian indices of the F1 (**A**) and F2 (**B**) treatment groups compared with those of the control group. In the first generation, rats in the low-dose (34.2 mg/kg) and high-dose (68.5 mg/kg) acephate treatment groups presented a significant increase in the ovarian index. However, no significant difference was found in the second generation. In the first generation (**C**), the number of offspring per female significantly decreased compared with that in the control group. In contrast, in the second generation (**D**), no significant difference in fertility rates was observed between the low-dose (34.2 mg/kg) group and the control group. In contrast, the high-dose acephate group (68.5 mg/kg) presented a lower fertility rate than the control group. Statistical significance was considered for all *p* values (* *p* < 0.05, **p< 0.01, *** *p* < 0.001, **** *p* value < 0.0001), ns: non significant.

The results of the present study indicated that females in the first generation from the two treatment groups (34.2 mg/kg and 68.5 mg/kg) had significantly greater ovarian indices (Figure 2A, *p* < 0.0001) than the control females did, suggesting that acephate may have an impact on ovarian development. Nevertheless, there were no notable effects on the ovarian indices of the females in the second generation (Figure 2B).

The number of offspring was counted to determine the impact of acephate on fertility. According to the findings, the fertility rate significantly decreased among females of the first generation, since the number of offspring was considerably reduced by exposure to both acephate doses (34.2 and 68.5 mg/kg) (Figure 2C). However, the significant decrease in the fertility rate among females in the second generation was significant only when the rats were exposed to the relatively high acephate dose (68.5 mg/kg) (Figure 2D).

### 3.3. Effect of Acephate on Ovarian Structure

**Figure 3:**
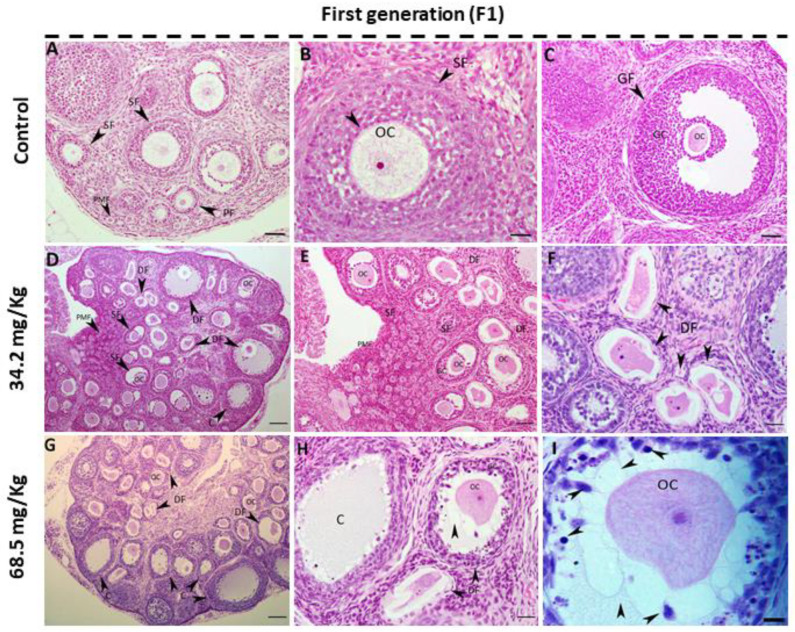
Photomicrographs of the H&E-stained ovarian sections. Panels (**A**–**C**) depict the control group, showing a typical ovarian histological structure with developing follicles. In contrast, panels (**D**–**I**) illustrate the ovaries of first-generation female rats exposed to acephate prenatally. A notable characteristic of these ovaries was the increased presence of degenerating follicles compared with those in the control group. These degenerated follicles typically exhibited abnormal oocytes and a reduced number of growing follicles. (**G**) In addition to the large number of degenerating follicles, there were ovarian cysts (arrowhead in (**G**)). (**H**,**I**) There was an appearance of many vacuoles localized around the oocytes, an unusual zona pellucida, and many pyknotic granulosa cell nuclei (arrowhead) in (**I**)). DF: degenerative follicle; GC: granulosa cell; OC: oocyte; PMF: primordial follicle; PM: primary follicle; SF: secondary follicle; C: cysts; GF: Graafian follicles. Scale bar = 20 µm.

While females from the control group presented a normal ovarian structure (Figure 3A–C), significant histological alterations were observed in the ovarian tissue of F1 females in the low- and high-dose treatment groups compared with the control group (Figure 3D–I). In particular, most of the growing follicles in the ovaries of first-generation female rats treated with both doses presented an altered architecture. The follicles of the ovaries from the 34.2 mg/kg acephate-treated F1 offspring had shrunken oocytes, probably leading to their degeneration (Figure 3D–F). Additionally, the ovaries from the 68.5 mg/kg acephate-treated F1 offspring presented with ovarian cysts in addition to a considerable number of degenerating follicles (Figure 3G–I). These degenerating follicles had increased numbers of pyknotic granulosa cell nuclei, uneven zona pellucida, and many large vacuoles around the oocytes (Figure 3H,I).

**Figure 4:**
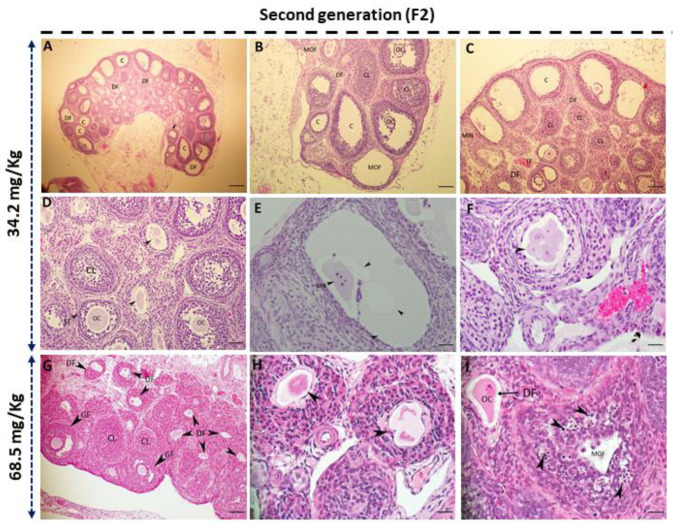
Microscopy images of the H&E-stained ovary tissue sections from second-generation female rats that were exposed (**A**–**I**). (**A**–**F**) Acephate treatment at 34.2 mg/kg resulted in many degenerating follicles, increased ovarian cysts, and many vacuoles (arrowheads in **E**). In addition, the micronucleus showed multioocyte formation (arrowhead in **F**). At a dose of 68.5 mg/kg, several corpus luteum, degenerating follicles (arrowheads in **H**)., and elevated GC pyknotic nuclei were detected (arrowheads in **I**). DF: degenerating follicle; OC: oocyte; SF: secondary follicle; TF: tertiary follicle; GF: Graafian follicles; C: cysts; CL: corpus luteum; MOF: multi oocyte formation; MN: micronucleus. Scale bar = 20 µm.

The ovaries from the F2 females presented an increased number of cysts and degenerating follicles (Figure 4A–C). Abnormal follicles containing fragmented oocytes or multiple oocyte–follicles were sometimes observed in the low-dose (Figure 4D–F) and high-dose treatment groups (Figure 4G–I).

### 3.4. Effect of Acephate on Cyp19 and Gdf9 Gene and Protein Expression

**Figure 5:**
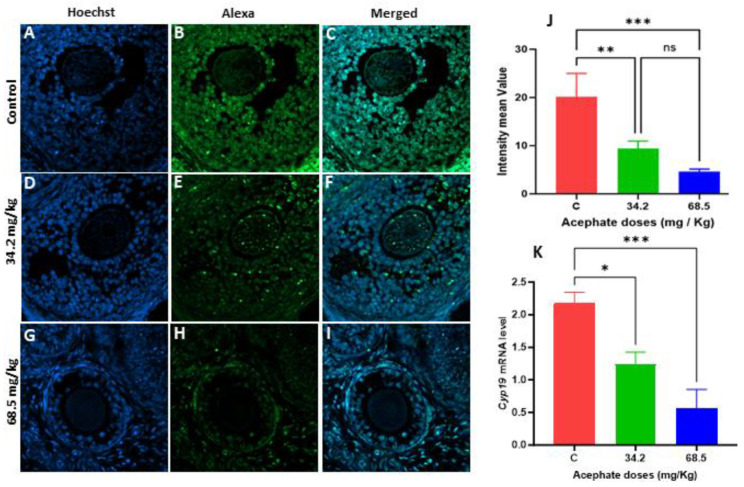
The localization of ovarian CYP19 in first-generation females (F1) was evaluated using immunofluorescence staining and visualized with a confocal microscope. (**A**–**C**) Control ovaries, (**D**–**F**) F1 ovarian acephate-treated ovaries (34.2 mg/kg), and (**G**–**I**) F1 ovarian acephate-treated ovaries (68.5 mg/kg). The comparative fluorescence intensity (**J**) of CYP19 in the control and treated groups was analyzed via Zen 3.1 software (ZEN lite) and quantified via GraphPad Prism 10 (version 10.1.2). Additionally, the mRNA levels of *Cyp19* in the F1 acephate-treated groups were compared with those in the control groups via qRT–PCR (**K**). (*) *p* value < 0.05, (**) *p* value < 0.01, (***) *p* value < 0.001, ns: non-significant.

**Figure 6:**
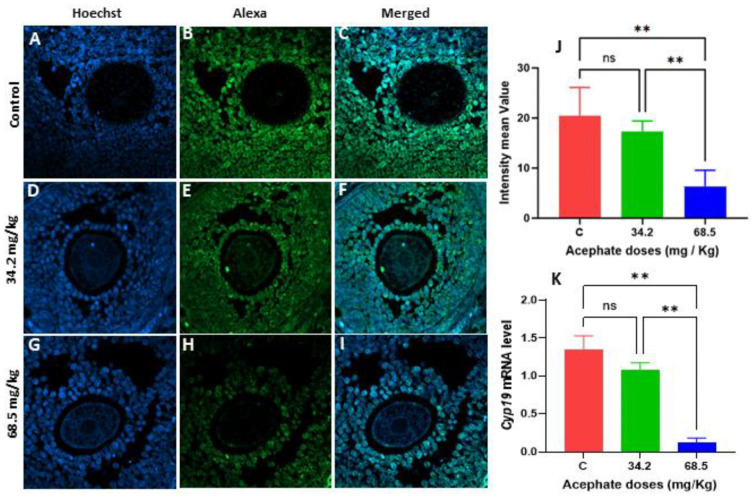
Localization and expression of CYP19 in ovarian tissue from second-generation females (F2). (**A**–**C**) Control ovaries, (**D**–**F**) F1 ovarian acephate-treated ovaries (34.2 mg/kg), and (**G**–**I**) F1 ovarian acephate-treated ovaries (68.5 mg/kg). Comparison of the fluorescence intensity of CYP19 in the control and exposure groups (**J**). The mRNA levels of *Cyp19* in the F2 acephate-treated groups were compared with those in the control groups via qRT–PCR (**K**) (**), *p* value < 0.01, ns: non-significant.

The protein and gene expression of *Cyp19* was assessed via immunofluorescence labeling and RT–PCR. The findings demonstrated that, in F1 females, the protein levels of CYP19 were significantly decreased in the 34.2 and 68.5 mg/kg treatment groups (Figure 5A–J). The same significant decrease was also observed in F2 females, but only in the 68.5 mg/kg acephate treatment group (Figure 6A–J). Similarly, the qRT-PCR results revealed that the *Cyp19* mRNA levels were significantly lower in both treatment groups of F1 females (Figure 5K). In contrast, in the F2 females, a significant decrease in the *Cyp19* mRNA levels was observed only in the high-dose treatment group (68.5 mg/kg) (Figure 5K).

**Figure 7:**
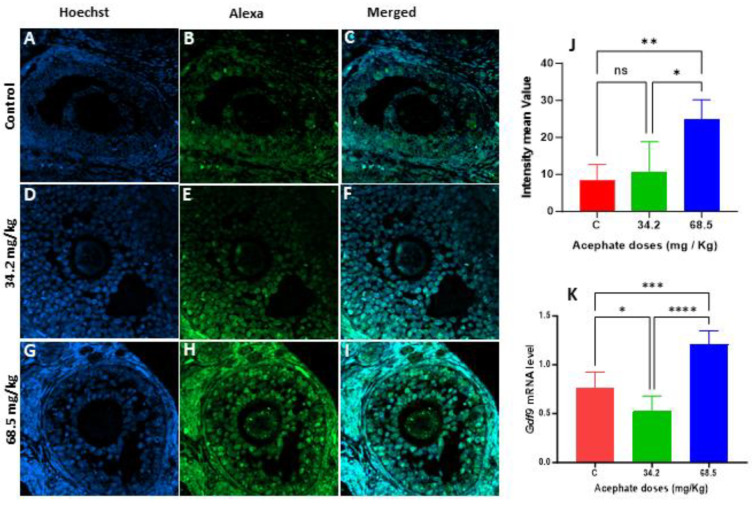
The localization of GDF9 in the ovarian tissue of first-generation females (F1) was assessed through immunofluorescence staining and visualized with a confocal microscope. (**A**–**C**) Ovaries from the control group, (**D**–**F**) F1 acephate-treated ovaries (34.2 mg/kg), (**G**–**I**) F1-treated acephate ovaries (68.5 mg/kg). Comparison of the fluorescence intensity of GDF9 in the control group and treatment groups (**J**). RT–PCR analyses of the mRNA levels of *Gdf9* in the first-generation acephate-treated groups compared with those in the control groups (**K**). (*) *p* value < 0.05, (**) *p* value < 0.01, (***) *p* value < 0.001, (****) *p* value < 0.0001, ns: non-significant.

**Figure 8:**
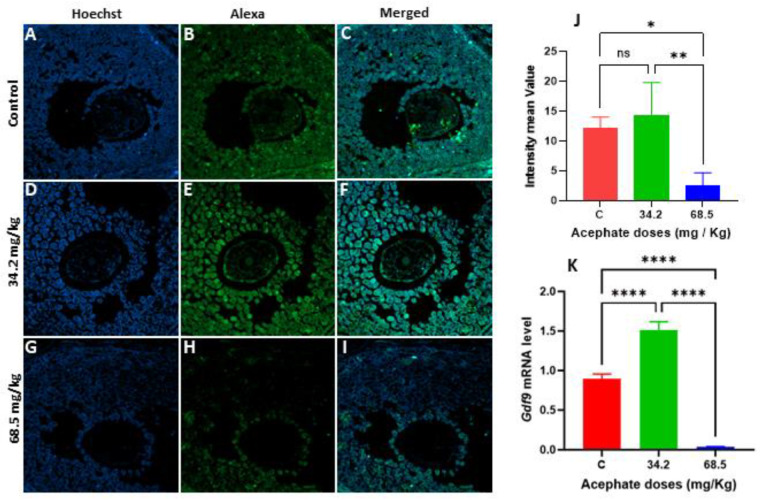
Illustration of the localization of GDF9 in ovarian tissue from females (F2), as assessed through immunofluorescence staining and visualized with a confocal microscope. (**A**–**C**) Ovaries from the control, (**D**–**F**) ovaries from 34.2 mg/kg acephate-treated F1, and (**G**–**I**) ovaries from first-generation females treated with 68.5 mg/kg acephate. Relative fluorescence intensity (**J**) of GDF9 in both the control and treatment groups. RT–PCR analyses of the *Gdf9* mRNA levels in the second-generation acephate-treated groups compared with those in the control groups (**K**). The scale bar represents 200 µm. Statistical significance is indicated by a (*) *p* value < 0.05, (**) *p* value < 0.01, (****) *p* value ≤ 0.0001, whereas ns denotes not significant.

In the first generation, the GDF9 protein levels were significantly elevated in the 68.5 mg/kg treatment group, whereas no significant differences were found in the females in the 34.4 mg/kg treatment group from the same generation, although the mRNA levels were decreased (Figure 7A–K). In contrast, the GDF9 protein level increased more in the 34.2 mg/kg treatment group in the second generation than in the other generations, whereas the GDF9 protein level strongly decreased in the 68.5 mg/kg treatment group in that generation (Figure 8A–K).

### 3.5. Impact of Acephate on the Expression of Genes Related to Folliculogenesis and Steroidogenesis

**Figure 9:**
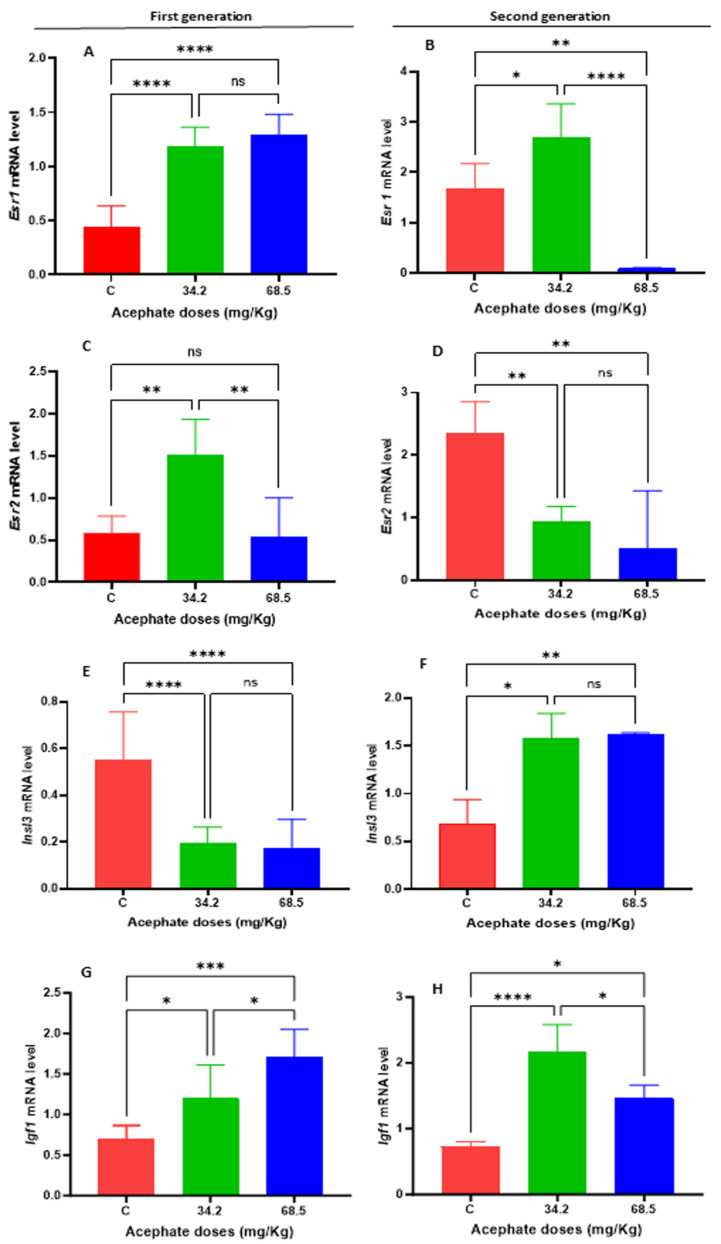
RT–PCR analyses of several genes related to folliculogenesis and steroidogenesis in the ovaries of rats from various treatment groups were performed, and the results were compared with those of the control group across multiple generations. (**A**) Changes in *Esr1* mRNA expression in the first generation. (**B**) Changes in *Esr1* mRNA expression in the second generation. (**C**) Changes in *Esr2* mRNA expression in the first generation. (**D**) Changes in *Esr2* mRNA expression in the second generation. (**E**) Changes in *Insl3* mRNA expression in the first generation. (**F**) Changes in *Insl3* mRNA expression in the second generation. (**G**) Changes in *Igf1* mRNA expression in the first generation. (**H**) Changes in *Igf1* mRNA expression in the second generation. (*) *p* value < 0.05, (**) *p* value < 0.01, (***) *p* value < 0.001, (****) *p* value < 0.0001, ns: Not significant.

To assess the impact of the increased expression of genes related to folliculogenesis and steroidogenesis, we examined the gene expression of *Esr1*, *Esr2*, *Insl3*, and *Igf1* (Figure 9). The mRNA levels of *Esr1* were significantly greater in the first-generation treatment groups, whereas there was a significant increase in the 34.2 mg/kg treatment group and a significant decrease in the 68.5 mg/kg treatment group in the second generation. Additionally, we detected a significant increase in the mRNA expression of *Esr2* in the 34.2 mg/kg treatment group in the first generation; however, we observed a substantial decrease in this response in the treatment groups in the second generation. The *Insl3* mRNA levels dramatically decreased in the first-generation 34.2 and 68.5 mg/kg treatment groups but significantly increased in the second-generation 34.2 and 68.5 mg/kg treatment groups. The *Igf1* mRNA levels significantly increased in the ovaries of both generations.

## 4. Discussion

Previous studies have indicated that processes that occur in the perinatal period can increase the risk of noncommunicable diseases including type 2 diabetes, cardiovascular disease, kidney failure, and infertility [3,39,40,41]. Since early life is a period of significant vulnerability for fetal and neonatal growth, the research community has raised concerns about the impact of environmental chemicals on fetal or neonatal development [42]. The use of drugs or other harmful chemicals during pregnancy and lactation continues to be a public health concern since they can stunt the growth of some fetal organs and/or tissues such as the ovary [10]. Unfavorable environmental stimuli during the embryonic stage or infancy may cause genetic reprogramming that alters tissue and/or organ function during the developmental stage [10]. These alterations, which can impact the health of future generations, can be maintained and even passed down to the next generation from adolescence to adulthood [33,43].

Our findings suggest that administering acephate to female rats resulted in a significant reduction in body weight among the F1 offspring, whereas there were no treatment-related effects on the body weights of the F2 offspring. This decline in body weight may be linked to metabolic impairments in pregnant mothers due to acephate exposure, which affected the metabolism of the F1 offspring while they are still in utero [44,45,46]. They were particularly vulnerable to metabolic disruptions during this rapid growth phase, which may have influenced their birth weight. In support of our results, previous studies have shown that early exposure to acephate is associated with low birth weight in offspring [3]. This condition can predispose offspring to various diseases in adulthood including type 2 diabetes, indicating that metabolic programming extends beyond nutritional factors to include other influences such as food contaminants such as acephate [3,17]. Since low birth weight can lead to alterations in lipid profiles as well as elevated triglyceride and insulin levels, F1 females may exhibit a normal phenotype while carrying an affected genotype that could be passed on to subsequent generations. Indeed, numerous studies have demonstrated a link between low birth weight and noncommunicable diseases [41,47].

Our findings revealed that the ovary weights of first-generation female rats that were administered acephate significantly increased. Although several studies have shown that organophosphate materials may affect female reproduction by lowering ovarian weights [48,49,50], our findings are consistent with previous studies showing that exposing pregnant female rats to certain compounds, such as fumonisin B1, acrylamide, sodium fluoride, BPA, and nonylphenol, results in increased ovarian weight [10,30,32,51,52]. Typically, ovarian weight in normal rats remains relatively stable, although it can fluctuate cyclically throughout the estrous cycle. Therefore, any notable increase or decrease in ovarian weight could be interpreted as a pathological case or a disruption in ovarian function, potentially linked to disorders such as reproductive aging, depletion of oocytes and follicles, persistent polycystic ovaries, and luteal cyst development [30]. This can be confirmed by the reduced fertility of F1 females compared with control females, as evidenced by the significant reduction in the number of offspring per female in the second generation. Moreover, previous studies have shown that the fertility rate decreases with increasing concentrations of many compounds such as glyphosate and acrylamide, or even as a result of increased environmental temperatures [53,54]. These chemicals can affect antioxidant defense, endocrine function, gametogenesis, and maturation [54]. The reduced fertility observed in F1 and F2 females in response to acephate exposure is partly a result of impaired folliculogenesis and/or steroidogenesis. Our examination of ovarian histoarchitecture revealed that exposure to various doses of acephate (34.2 and 68.5 mg/kg) altered the ovarian structure of the F1 offspring, which was characterized mainly by shrunken and vacuolized oocytes, probably leading to their degeneration given the increased number of pyknotic granulosa cell nuclei and uneven zona pellucida. Similarly, ovaries from F2 females presented an increased number of degenerating follicles and abnormal follicles sometimes containing fragmented oocytes or multioocyte follicles. Moreover, cyst formation was noted in the ovaries of the high-dose treatment groups (68.5 mg/kg), which may be interpreted as indicative of luteinized unruptured follicle syndrome [55]. These adverse histological effects induced by acephate exposure could explain the reduced fertility among the two generations. Thus, the ovaries of the second-generation treated rats appeared grossly similar in size to those of the control, but histologically, they were quite altered.

One key enzyme examined in this study was CYP19 aromatase, which facilitates the conversion of androgens to estrogens. In line with the histopathological findings, we observed that CYP19 protein expression was significantly reduced in both treated groups of the F1 offspring, which was confirmed by a notable decrease in the mRNA levels of the *Cyp19* gene measured via qRT–PCR. Given that this enzyme is essential for female reproduction, its decline undoubtedly adversely affects the fertility of F1-generation females, as shown by ovarian histological analysis. These findings align with those of previous studies, indicating that exposure to certain organophosphate chemicals can lead to reduced aromatase activity [56,57,58]. In fact, several pesticides including imazalil, prochloraz, fenarimol, triadimenol, triadimefon, and dicofol have been shown to significantly inhibit aromatase activity, thereby impacting the sex hormone levels and the development of the female reproductive system [56]. Interestingly, decreased levels of the *Cyp19* gene and protein expression were also detected in the F2 generation, which was in accordance with the findings in the F1 generation. These results confirm that acephate exerts a transgenerational inheritance effect on ovarian developmental toxicity along with associated changes in reproductive phenotype.

GDF9, which is secreted by oocytes, plays a crucial role in ovarian follicular development [59]. It regulates the recruitment of primordial follicles, increases the proliferation of granulosa cells, inhibits their apoptosis, promotes oocyte maturation, and promotes steroid hormone synthesis [60]. In our study, we found that acephate increased both *Gdf-9* mRNA and protein expression in the ovaries of F1 females from the high-dose treatment group. These elevated levels of GDF9 suggest a disruption in normal follicular development that warrants further investigation. Previous reports have also shown that the upregulation of *Gdf-9* is associated with follicular atresia following treatment with the neurotoxin 3,3-iminodipropionitrile [61]. Similarly, studies have shown that early follicle development in rat ovaries irradiated during the neonatal period results in oocytes that strongly express GDF9, which is linked to premature ovarian failure [62]. In these cases, primordial follicles are depleted, and the cuboidal granulosa cells in primary-like follicles do not proliferate. The upregulation of *Gdf-9* expression has been interpreted as an early response from oocytes attempting to compensate for granulosa cell damage in atretic follicles [63]. Thus, the increased levels of GDF-9 in F1 females reflect ovarian impairment due to acephate treatment. Although the precise mechanism of action remains unclear, consistent with previous studies, we hypothesize that elevated *Gdf-9* levels are a response to induced follicular atresia, as confirmed by the histopathological findings. In F2 females from the 68.5 mg/kg treatment group, the *Gdf-9* levels were significantly reduced, likely indicating infertility, which is in line with previous research [64]. Since F1 females exhibited increased GDF9 levels at a high dose, F2 females may anticipate this intensive increase by trying to adjust the GDF9 levels using an unknown mechanism, leading to its downregulation, which is consistent with our previous reports [38].

The primary mechanism of action of estrogen involves the expression of the nuclear estrogen receptors *Esr1* and *Esr2*, which exhibit different expression profiles in various tissues [65]. ESR1 is found in all three components of the hypothalamic–pituitary–ovarian axis, whereas ESR2 is prominent in ovarian granulosa cells. This distinct expression pattern suggests that each receptor plays a separate role in regulating ovarian function. Research involving female ESR1 knockout (ERK1O) mice indicates that ESR1 is essential for the feedback effects of estradiol, which regulate proper luteinizing hormone (LH) secretion from the pituitary, whereas ERS2 is not [66]. ESR1 is widely accepted to mediate estrogen feedback actions on GnRH/LH release through afferent neurons expressing ESR1, particularly the kisspeptin neurons responsible for KISS1 production [65]. Given our findings of a dose-dependent decrease in CYP19 among F1 and F2 females with both treatments—likely leading to reduced estrogen production—we anticipated a corresponding reduction in the expression of *Esr1* and Esr2 among the respective groups. However, the observed disruptions in *Esr1* and *Esr2* do not align with aromatase activity, suggesting that acephate may affect ovarian function through a different mechanism rather than solely through estrogen receptors. Similar to our findings, the metabolite monoethylhexyl phthalate (MEHP) causes decreased granulosa cell aromatase RNA expression and protein levels in a dose-dependent manner, which consequently results in decreased serum estradiol levels and no ovulations in adults [67]. The authors concluded that phthalates suppress aromatase activity and estradiol production through the activation of peroxisome proliferator-activated receptors (PPARs) but do not act via the estrogen receptor [67,68]. However, any dysregulation of gonadotropin secretion linked to a failure in follicle growth and ovulation is caused by the disruption of either *Esr1* or *Esr2* signaling, indicating that an initial ovarian abnormality is caused by the inhibition of estrogen signaling [69,70]. The effects of pesticides on the ovary and follicles that have been observed could be the result of an imbalance in hormones caused by a direct influence on the ovary or the hypothalamic–pituitary–ovarian axis [71]. Indeed, pesticide exposure has been shown to cause decreased ovulation and fertility as well as increased follicular atresia in rats. This may be because pesticides can behave as endocrine disruptors and may change ovarian gene expression and folliculogenesis by lowering the serum progesterone levels [72,73].

*Insl3* is a key regulator of female reproductive physiology, is produced primarily by theca interna cells in growing antral follicles, and serves as an indicator of follicle number and health [74]. It is also the main steroid precursor for granulosa cells to produce estrogen. Our data indicate that Insl3 was downregulated in the F1 generation, which is consistent with histopathological findings showing a significant number of disrupted follicles. These findings suggest that early-life exposure to acephate may inhibit the growth of healthy follicles by inhibiting *Insl3* expression in the ovaries of the offspring. Knockout studies in mice have confirmed that the absence of *Insl3* or its receptor in females results in partial infertility, marked by a decrease in follicle numbers, ovulations, and litter size. Interestingly, the mRNA levels of the *Insl3* gene were greater in the F2 offspring than in the control offspring. Since this gene is known for being expressed at elevated levels in the premenopausal ovary and in women with PCOS [74], acephate may contribute to cyst formation, as indicated by our histological examination [39]. Thus, acephate may exert a direct short-term effect on *Insl3* expression in F1 females by inhibiting ovarian function while also having an indirect long-term effect on F2 females by stimulating Insl3 release, potentially leading to a PCOS phenotype [75].

*Igf1* expression is closely associated with the growth of ovarian follicles and the proliferation of granulosa cells in prepubertal female ovaries [76]. It promotes the release of steroid hormones including estradiol and progesterone, resulting in ovarian cell proliferation [77]. Insufficient *Igf1* activity in rat granulosa cells is associated with reduced steroidogenic enzyme expression and circulating estrogen levels, along with abnormal ovarian development [78]. We observed that the mRNA level of the *Igf1* gene was significantly greater in the F1 generation than in the control group. Research has shown that exposure to endocrine disruptors (EDCs) can lead to early puberty onset, characterized by increased levels of *Igf1* and GnRH, along with earlier vaginal opening and activation of the IGF-1/PI3K/AKT/mTOR pathway [79]. Similarly, we propose that acephate may accelerate puberty onset in prepubertal F1 female rats, with this accelerated onset potentially being inherited by the F2 generation, as shown by the increase in *Igf1* gene expression in the ovaries of F2 females. This finding aligns with previous epidemiological studies that have linked precocious puberty in girls to elevated levels of *Igf1* in peripheral blood [80,81]. Moreover, exposure to endocrine disruptors during pregnancy and lactation has been shown to induce early puberty in the female offspring of rats, affecting genes related to reproductive development such as adiponectin receptor 1 (*AdipoR1*) and *Igf1*, which may contribute to female reproductive endocrine disorders [82]. Furthermore, studies have indicated that the consumption of foods high in DEHP during pregnancy is positively correlated with increased serum IGF1 levels in girls aged 8–14 years [83].

## 5. Conclusions

The results of the present study demonstrated that exposure to acephate during fetal development leads to ovarian developmental toxicity in first-generation females. The affected ovaries are primarily characterized by structural abnormalities, decreased fertility, early onset of puberty, and impaired expression of folliculogenesis and steroidogenesis markers. Second-generation females presented reproductive phenotype alterations similar to those of F1 females, providing evidence of transgenerational inheritance. In addition to altered ovarian markers, reduced fertility, and early puberty, second-generation females presented a phenotype resembling PCOS. The mechanisms underlying this toxicity may be associated with changes in the ovarian *Cyp19*, *Gdf9*, *Insl3*, and *Igf1* levels. Further research is needed to evaluate the reproductive health risks linked to acephate exposure.

## Data Availability

The data that support the findings of this study are available from the corresponding author (Abdel Halim Harrath) upon reasonable request.

## Supplementary Materials

The following supporting information can be downloaded at: https://www.mdpi.com/article/10.3390/biology13121075/s1, Table S1: Primers for the real-time RT–PCR.
