# Supplementary material for: Acephate Exposure Induces Transgenerational Ovarian Developmental Toxicity by Altering the Expression of Follicular Growth Markers in Female Rats
Source: Biology (Basel). 2024 Dec 20;13(12):1075. doi: 10.3390/biology13121075 (PMC11673910; doi:10.3390/biology13121075)
Supplement: Supplementary file 1 [file biology-13-01075-s001.zip › biology-3352227-supplementary.pdf]

Table S1. Primers for the real-time RT–PCR.

Gene Symbol	Sequences
**Esr1**	Forward: C A T C G A T A G A C G A G A<br>Reverse: A G T G C A G C T C T C A T G T
**Esr2**	Forward: G A G C T G A C A C C A T G T<br>Reverse: C A G T C C A C A T A G C A C T
**Igf-I**	Forward: C G C T G A G C T A C A A G T C<br>Reverse: G G A G C T C T C T A C A T C
**Insl3**	Forward: C T C T C A C A G C T C T C A<br>Reverse: C A C A C T G A G C C T A C A T
**Cyp19**	Forward : GCAACAGGAGCTATAGATGAAC<br>Reverse : AGGCACGATGCTGGTGATG3
**Gdf9**	Forward : GATGTGACCTCCCTCCTTCA<br>Reverse : GCCTGGGTACTCGTGTCATT
